# Rnd3 Expression is Necessary to Maintain Mitochondrial Homeostasis but Dispensable for Autophagy

**DOI:** 10.3389/fcell.2022.834561

**Published:** 2022-06-27

**Authors:** Cristina Cueto-Ureña, Enric Mocholí, Josep Escrivá-Fernández, Susana González-Granero, Sabina Sánchez-Hernández, Amalia Solana-Orts, Begoña Ballester-Lurbe, Karim Benabdellah, Rosa M. Guasch, José Manuel García-Verdugo, Francisco Martín, Paul J. Coffer, Ignacio Pérez-Roger, Enric Poch

**Affiliations:** ^1^ School of Health Sciences, Universidad CEU Cardenal Herrera, CEU Universities, Valencia, Spain; ^2^ Center for Molecular Medicine, University Medical Center Utrecht, Utrecht, Netherlands; ^3^ Laboratorio de Neurobiologia Comparada, Instituto Cavanilles de Biodiversidad y Biologia Evolutiva, Universidad de Valencia and CIBER de Enfermedades Neurodegenerativas (CIBERNED), Valencia, Spain; ^4^ Genomic Medicine Department, GENYO, Centre for Genomics and Oncological Research, Pfizer-University of Granada-Andalusian Regional Government, Health Sciences Technology Park, Granada, Spain; ^5^ Departamento de Bioquímica y Biología Molecular, Facultad de Farmacia, Universidad de Valencia, Valencia, Spain

**Keywords:** Rnd3/RhoE, neurodisorders, mitochondrial dysfunction (MtD), autophagy, OXPHOS (oxidative phosphorylation)

## Abstract

Autophagy is a highly conserved process that mediates the targeting and degradation of intracellular components to lysosomes, contributing to the maintenance of cellular homeostasis and to obtaining energy, which ensures viability under stress conditions. Therefore, autophagy defects are common to different neurodegenerative disorders. Rnd3 belongs to the family of Rho GTPases, involved in the regulation of actin cytoskeleton dynamics and important in the modulation of cellular processes such as migration and proliferation. Murine models have shown that Rnd3 is relevant for the correct development and function of the Central Nervous System and lack of its expression produces several motor alterations and neural development impairment. However, little is known about the molecular events through which Rnd3 produces these phenotypes. Interestingly we have observed that Rnd3 deficiency correlates with the appearance of autophagy impairment profiles and irregular mitochondria. In this work, we have explored the impact of Rnd3 loss of expression in mitochondrial function and autophagy, using a Rnd3 KO CRISPR cell model. Rnd3 deficient cells show no alterations in autophagy and mitochondria turnover is not impaired. However, Rnd3 KO cells have an altered mitochondria oxidative metabolism, resembling the effect caused by oxidative stress. In fact, lack of Rnd3 expression makes these cells strictly dependent on glycolysis to obtain energy. Altogether, our results demonstrate that Rnd3 is relevant to maintain mitochondria function, suggesting a possible relationship with neurodegenerative diseases.

## Introduction

Neurodegenerative diseases are complex entities characterized by a gradual loss of neuronal function. In some of these diseases, including Parkinson’s disease (PD), Alzheimer’s disease (AD), Huntington’s Disease (HD) and Amyotrophic Lateral Sclerosis (ALS), common pathological features are the accumulation of aggregate-prone proteins that causes neuronal degeneration (Menzies et al., 2017) and an imbalance of mitochondrial homeostasis characterized by decreased activity of respiratory chain enzymes and abnormal morphology (Xu et al., 2021).

Autophagy is a cellular process that involves the degradation of internal components through the lysosomal machinery and that has been proposed as a potential therapeutic target for neurodegenerative disorders (Scrivo et al., 2018). In the Central Nervous System (CNS), autophagy eliminates defective proteins and organelles, preventing the accumulation of aggregates, providing metabolites to accommodate the neuronal energy demands, and supporting neuronal plasticity (Guo et al., 2018; Park et al., 2020). Mitophagy, a selective form of autophagy, is also crucial for the removal of nonfunctional mitochondria in the cell. Therefore, autophagy/mitophagy impairment has been involved, directly or indirectly, in numerous neurological disorders (Schneider and Cuervo, 2014; Menzies et al., 2015), whereas autophagy activation has been demonstrated to be essential to prevent neuronal function damage and aging (Pyo et al., 2013; Hansen et al., 2018).

Rho proteins are monomeric small GTPases that have been classically described as regulators of the actin cytoskeleton dynamics and cell migration (Hansen et al., 2018). Some of these proteins have also been linked to a wide range of neuronal functions and some neurodegenerative disorders (Zhang et al., 2021). For instance, Rac1 is able to activate APP (Amyloid Precursor Protein) gene transcription (Wang et al., 2009) resulting in an aberrant accumulation of extracellular amyloid-β (Aβ) plaques in AD. RhoA is dysregulated in brains from human AD patients and mice models (Petratos et al., 2008; Huesa et al., 2010) and inhibition of the RhoA effector ROCK-I promotes survival of dopaminergic neurons and also attenuates axonal loss in a PD mouse model (Tonges et al., 2012).

Rnd3 is a member of the Rnd subfamily of Rho GTPases that negatively regulates the RhoA-ROCK-I-MLC pathway (Jie et al., 2015). Rnd3 is ubiquitously expressed in adult brain, with high expression levels during postnatal development that decrease in adulthood (Ballester-Lurbe et al., 2009). We and others have demonstrated an essential role of Rnd3 in the CNS development. Mice lacking Rnd3 expression are significantly smaller and have a shorter lifespan than wild-type mice (Mocholi et al., 2011). Rnd3 deficiency produces severe motor alterations as well as a delay in the neural behavior development and causes a decrease in axon outgrowth and a delay in neuronal polarization (Peris et al., 2012). In addition, Rnd3 has an essential role in cell migration along the rostral migratory stream (RMS) and is crucial for proper olfactory bulb cell development (Ballester-Lurbe et al., 2015). Finally, we have recently described that Rnd3 is necessary for the formation of dendritic spines and the corticospinal axon pathfinding as well as in the globus pallidus formation (Kaur et al., 2020; Marfull-Oromi et al., 2021). These and other evidence point out the important role of Rnd3 in neurodevelopment, although the molecular mechanisms involved are still poorly understood.

As actin cytoskeleton participates in autophagy, Rho proteins could act as regulators of this process. Overexpression of Rac3 produces a decrease in microtubule-associated protein 1A/1B-light chain 3B (LC3), an essential protein for autophagosome formation (Zhu et al., 2011). Rac1 inhibits the increase of LC3 levels in its active form (Aguilera et al., 2012), but is necessary for chaperone-mediated autophagy (CMA) (Arias et al., 2015). Active RhoA induces autophagy, reaching LC3 levels similar to those obtained under starvation (Aguilera et al., 2012). ROCK-I has also been involved in neurodegeneration (Koch et al., 2018), but its role in autophagy remains unclear. Inhibition of ROCK-I produces a decrease in LC3 levels, but it has no effect on autophagy in the presence of active RhoA (Aguilera et al., 2012); however, it promotes autophagic vesicles formation under starvation (Gurkar et al., 2013; Mleczak et al., 2013).

Although active Rho GTPases have been traditionally proposed to reside and function predominantly on the plasma membrane, it has been established that some of them are also localized to endomembranes, such as endosomes, Golgi and mitochondria (Phuyal and Farhan, 2019). Mitochondria are key components of the cellular machinery, sitting at the interface of cellular metabolism, energy production, and cell death. Regarding neural development, mitochondria have been considered a signaling platform which allows an increase in the production of ATP necessary for many processes such as neurite growth, neuronal polarity and plasticity (Cheng et al., 2010).

As Rnd proteins, the mitochondrial Rho proteins (Miro) are atypical GTPases and are involved in mitophagy and mitochondrial quality control as well as in mitochondrial axonal transport and mitochondrial dynamics (Fransson et al., 2003; Eberhardt et al., 2020; Panchal and Tiwari, 2021). Rac1, but not RhoA or Cdc42, was also detected in mitochondria-enriched fractions from several tissues, including neuronal cells (Natsvlishvili et al., 2015) and Rac1 signaling was proposed to play important roles in the regulation of neuroplasticity and mitochondrial oxidative stress (Pan et al., 2018), although its function remains to be clarified.

In this work, we analyzed the possible role of Rnd3 in autophagy and mitochondrial function. Our results show that mice lacking Rnd3 expression display autophagy disfunction profiles and irregular mitochondria. Rnd3 KO cells in culture show no defects in autophagy but have impaired mitochondrial metabolism and are strictly dependent on glycolysis, which could explain some of the neural phenotypes seen in Rnd3 KO mice. Our results suggest that Rnd3 is relevant for mitochondrial metabolism and could be involved in the onset or progression of some neurodegenerative diseases.

## Materials and Methods

### Animal Procedures

Mice deficient for Rnd3 expression (Rnd3^gt/gt^) were generated at Lexicon Pharmaceuticals. The resulting phenotype has been described previously (Mocholi et al., 2011). Mice were handled according to European Union and National rules. All procedures were conducted in accordance with the guidelines of the Institutional Animal Care (CEEA 19/004).

### Cell Culture

Rnd3 deficient cells (Rnd3 KO) were obtained from NIH 3T3 murine fibroblasts using CRISPR/Cas9 gene editing technology. Cells were cultured at 37°C and 5% CO_2_ in Dulbecco’s modified Eagle’s medium supplemented with 10% fetal bovine serum and 1% penicillin/streptomycin (Gibco, Invitrogen). We used CRISPOR (CRISPOR.org) to design two different guide RNAs (gRNA6: GTA​AAT​CTA​TCA​TGG​ATC​CTT​GG and gRNA1: GTA​GTG​GGC​GAC​AGC​CAG​TGT​GG) in the first exon of RND3. gRNAs were synthesized by GenScript and cloned in the pUC57 vector under the U6 promoter control. NIH 3T3 cells were cotransfected with the above constructs and CESC9, which contains the Cas9 endonuclease and the green fluorescent protein (eGFP). 48 hours post-transfection, eGFP positive cells were sorted by flow cytometry and serial dilutions were plated to obtain single cell clones. RND3 editing was confirmed by DNA sequencing and Western blot.

Mouse embryonic fibroblasts (MEF) were obtained as previously described (Hernández-Sánchez et al., 2015) and immortalized with the SV40 Large Tumor antigen (SV40-LT) expressing retrovirus.

### Electron Microscopy

Brains from 15 days old wildtype and Rnd3^gt/gt^ mice (P15) were removed from the braincase after cervical dislocation and fixed overnight in 2% paraformaldehyde/2.5% glutaraldehyde, as previously described (Madrigal et al., 2021). Ultra-thin (0.07 μm) sections corresponding to the subventricular zone (SVZ) were obtained for the corresponding ultrastructural analysis.

3T3 and MEF cells were seeded on 8-well Permanox chamber slides (Nunc, Thermo Scientific) and were subsequently fixed in tempered 3.5% glutaraldehyde in 0.1 M phosphate buffer (PB) for 10 min at 37 °C. Cells were postfixed in 2% OsO4 for 1 h at room temperature, rinsed, dehydrated and embedded in Durcupan resin (Fluka, Sigma-Aldrich). Later ultra-thin (70 nm) sections of the cells were cut with a diamond knife, stained with lead citrate (Reynolds solution).

All samples were examined under a transmission electron microscope (FEI Tecnai G2 Spirit BioTwin) with a Xarosa (20 Megapixel resolution) digital camera using Radius image acquisition software (EMSIS GmbH, Münster, Germany).

For the *in vivo* analysis, subventricular zone (SVZ) brain samples from three mice of each genotype were analyzed. Micrographs were taken at 3 levels (anterior-posterior axis) of the SVZ and the density of mitochondria, lysosomes, lipid droplets was analyzed. For the *in vitro* analysis, three independent cultures of each cell line and genotype were studied and the mitochondria of 10 cells were analyzed.

### Western Blot Analysis

Protein extracts from tissue and cells were obtained as previously described (Hernández-Sánchez et al., 2015). Membranes were probed with the corresponding primary antibodies: anti-Rnd3 (05–723), anti-β-Tubulin (4026) and anti-β-Actin (A-3854), from Sigma; anti-PDI (2446), anti-Tom20 (42406) and anti-LC3B (2775), from Cell Signaling; anti-Hsp60 (386029), from Calbiochem. After incubation with the appropriate secondary antibodies, blots were revealed, and bands were quantitated using Image J software.

### Proliferation and Viability Assays

For proliferation assays, Rnd3 WT and KO cells were plated on 6 cm culture dishes. Every 48 h, starting 24 h after plating (time 0), cells were stained with trypan blue (Sigma) and counted in a Neubauer chamber.

For viability assays, Rnd3 WT and KO cells were plated on 12-well dishes. Cells were treated with 2-deoxy-D-Glucose (2-DG, 50mM, Sigma) for 4, 6, and 16 hours. After treatment, cells were collected and counted as described above. Results were represented as the percentage of viable cells compared to untreated cells.

### Analysis of Autophagy

Rnd3 WT and KO cells were plated on 6-well culture dishes and cultured in the presence or absence of serum for 4 or 6 hours. Cells were treated with inhibitors of lysosomal proteolysis (20 mM NH_4_Cl and 200 μM leupeptin, PI) for either 2 or 4 hours to block protein degradation. LC3-II and other autophagy markers were analyzed by Western blot. Image J software was used to quantify band intensity. Total protein in each lane was quantified after staining with 0.1% Ponceau S (Sigma) and used as loading control.

### Analysis of Mitophagy and Evaluation of Mitochondrial Functionality by Flow Cytometry

Rnd3 WT and KO cells were plated on 6-well culture dishes. Carbonyl cyanide 4-(trifluoromethoxy) phenylhydrazone (FCCP, 10 µM. Sigma) treatment for 16 hours was used to induce mitochondrial damage and degradation. To block mitophagy, the lysosomal inhibitor hydroxychloroquine (HCQ, 30 μg/ml, Sigma) was added 5 hours before FCCP treatment. To evaluate total mitochondrial mass, cells were stained with 100 nM Mitotracker Green FM (MTGreen, Thermo Fisher Scientific) for 30 min in the dark at 37°C and 5% CO_2_. Cells were washed twice with 1x PBS and the mean fluorescence intensity (MFI) was measured by flow cytometry (CytoFlex, Beckman Coulter). The results were represented as the mean fluorescence intensity (MFI) of MTGreen.

To analyze the integrity of the mitochondrial membrane potential, cells were treated with FCCP for 15 or 30 min. To measure total active mitochondria, cells were stained with 200 nM Tetramethylrhodamine methyl ester (TMRM, Thermo Fisher Scientific) and total mitochondrial mass was evaluated with MTGreen as described above. Staining was performed in the dark at 37°C and 5% CO_2_ for 30 min. Cells were washed and the fluorescence intensity was measured as described above. The results were expressed as the TMRM/MTGreen rate, which corresponds to healthy/active mitochondria.

### Assessment of Mitochondrial Respiration and Glycolysis

Oxygen consumption rate (OCR) and extracellular acidification rate (ECAR) were measured in Rnd3 WT and KO cells with an XF24 Extracellular Flux Analyzer (Seahorse Bioscience) using a cell mito stress test kit and a glycolysis stress test kit, respectively. Each cell line was plated in triplicates on XF 24-well cell culture microplates (Seahorse Bioscience). Where indicated, 750 µM H_2_O_2_ was added for 3 hours to induce oxidative stress. For OCR assays, cell growth medium was replaced by Seahorse XF DMEM medium (Agilent) supplemented with 1 μM glucose, 1 mM pyruvate and 2 mM glutamine and incubated for 1 h in a CO_2_ free incubator prior to the assay. For ECAR analysis, growth medium was replaced by Seahorse XF DMEM medium supplemented with 1 mM pyruvate and 2 mM glutamine and cells were preincubated at 37°C without CO_2_ for 1 h, before starting the assay. After baseline measurements, OCR was measured after sequentially adding to each well oligomycin, FCCP and rotenone, to reach working concentrations of 1, 5, and 1μ M, respectively. ECAR was measured after baseline measurements and after sequentially adding to each well glucose, oligomycin and 2-DG to reach working concentrations of 30 mM glucose, 1 μM oligomycin and 100 mM 2-DG.

Mitochondria respiration (basal respiration, maximal respiration, proton leak and ATP production) and glycolysis were analyzed according to the XF cell mito stress and XF cell glycolysis stress test kit User Manuals, respectively.

### Statistics

Values represent mean ± SEM. All statistical analysis were performed using GraphPad Prism (GraphPad Software). Statistical comparison among groups was evaluated using one-way ANOVA and a Bonferroni’s *post-hoc* test, an unpaired *t* test or a Kolmogorov-Smirnov *t* test, as indicated. In all cases, *p* < 0.05 was considered statistically significant.

## Results

**FIGURE 1:**
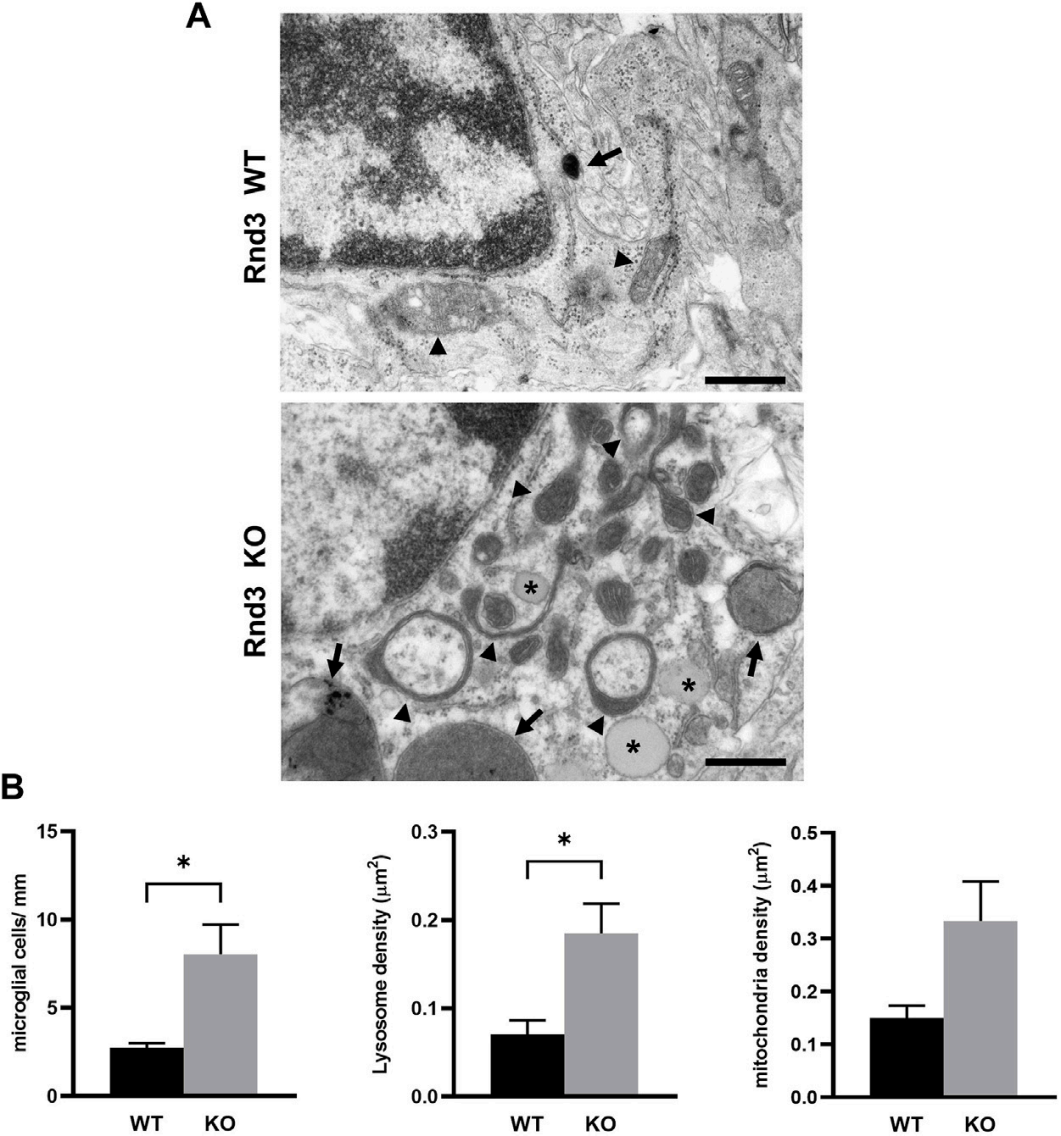
Lack of Rnd3 expression causes ultrastructure alterations in the subventricular zone (SVZ). **(A)** Electron microscopy (EM) images of microglial cells from Rnd3 deficient mice (Rnd3 KO, bottom panel) showing numerous lysosomes (arrows) and mitochondria, some of them with irregular morphologies (arrowheads) and lipid droplets accumulation (asterisks), when compared with wildtype samples (Rnd3 WT, top panel). Scale bar: 500 nm. **(B)** Quantitative analysis of the EM study of the subventricular zone (SVZ) of wildtype (WT) and Rnd3 deficient (KO) mice. Microglial cells were counted in three different levels of the SVZ (left), as described in Methods. Lysosome (middle) and mitochondria (right) density represent the number of either lysosomes or mitochondria per cell area. Plots represent the mean and standard error of the mean (SEM) of three independent samples (microglial cells) or all the microglial cells found (organelle density; WT, n= 23 cells; Rnd3^gt/gt^, n = 43 cells). No lipid droplets were found in WT samples and therefore this is not shown in the graphs. Unpaired *t* test shows statistical differences in the number of microglial cells (*p* = 0.0364) and lysosome density (*p* = 0.0196).

Our previous results demonstrated that Rnd3 deficient mice show alterations both in the structure and the function of the CNS (Mocholi et al., 2011; Ballester-Lurbe et al., 2015; Kaur et al., 2020; Madrigal et al., 2021; Marfull-Oromi et al., 2021). To determine if there were defects also at the subcellular level, we analyzed brain sections at the subventricular zone (SVZ) by electron microscopy (Figure 1A), where we had previously described several alterations (Ballester-Lurbe et al., 2015). Rnd3 deficient mice show a higher number of microglial cells compared to wild-type mice (Figure 1B). Additionally, these cells show an accumulation of lysosomes and also lipid droplets that cannot be found in wild-type samples (with an average density of 0.056 lipid droplets/μm^2^ or 2.37 lipid droplets per cell in mutant mice). Finally, there is a non-significant increase in the number of mitochondria (Figure 1B), and some of them show an elongated morphology and expanded intermembrane spaces (Figure 1A). In order to see whether Rnd3 is associated with mitochondria, we analyzed subcellular fractions by western blotting. Rnd3 was found in membranes, as previously described (Madigan et al., 2009) but not in the mitochondria enriched fraction (Supplemental Figure S1). The alterations seen in brains from Rnd3 deficient mice may be indicative of autophagy problems and suggest that Rnd3 could have a role in mitochondrial homeostasis.

**FIGURE 2:**
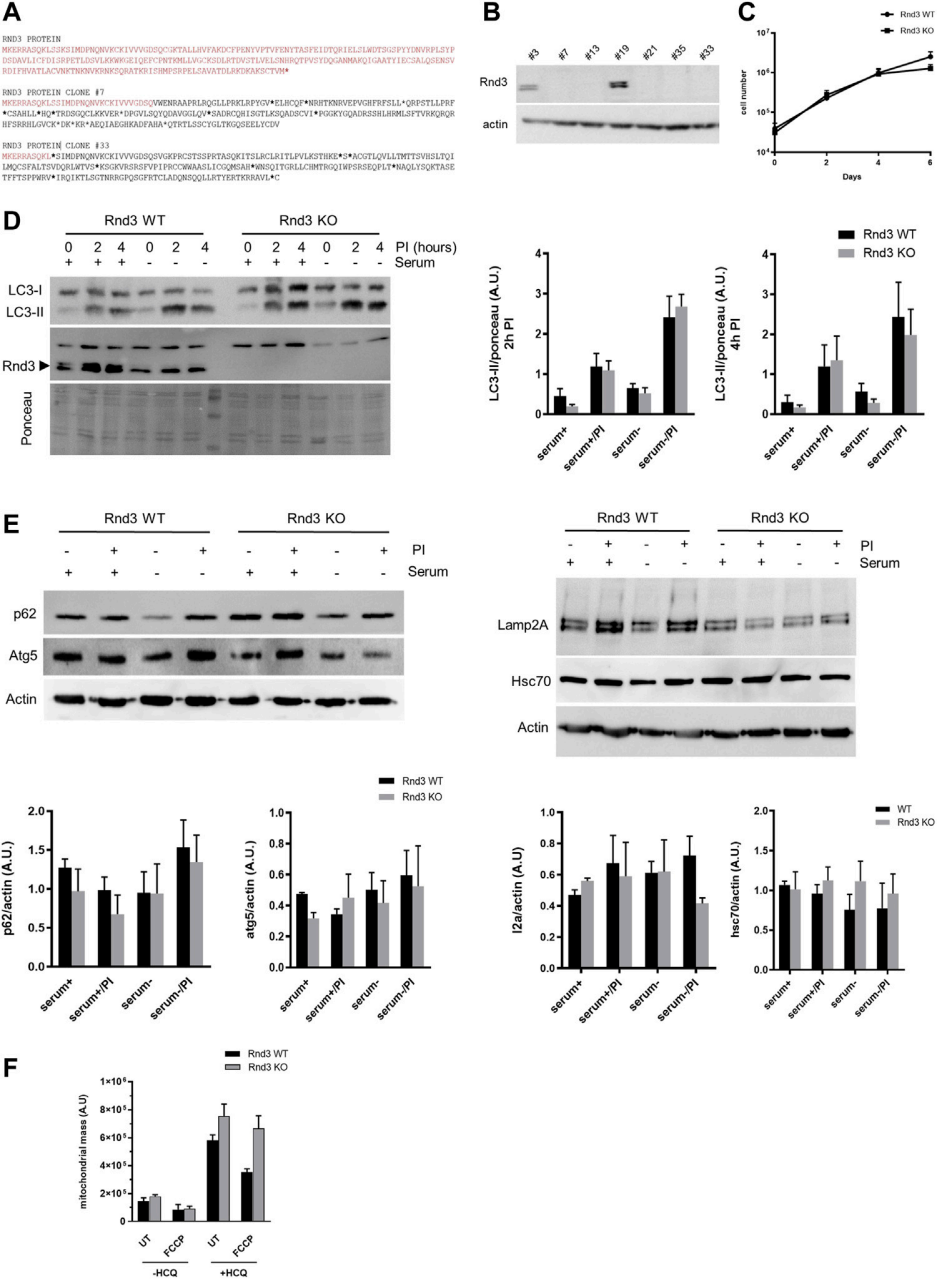
Autophagy and mitophagy are not altered in cells lacking Rnd3 expression. **(A)** Representative aminoacidic sequences from CRISPR Rnd3 edited cells obtained by single cell cloning (clones #7 and #33) Red letters correspond to wild-type Rnd3 protein and blackletters correspond to the edited sequences. Asterisks represent STOP codons. **(B)** Rnd3 expression was analyzed by Western blot in different clones. Clone #33, showing no expression of Rnd3, was selected for further analysis (Rnd3 KO). **(C)** Rnd3 KO cells have no defects in cell growth. Rnd3 WT and KO cell cultures were analyzed as described in Methods. **(D)** Autophagy is not impaired in Rnd3 KO cells. Autophagy flux was analyzed in the presence or absence of serum. Inhibitors of lysosomal proteolysis (PI) were added to block lysosomal protein degradation. LC3-II levels were analyzed by Western blot (left) as described in Methods and its relative levels are plotted (right panel, n = 5). **(E)** Analysis of autophagy markers. Cells were treated as in **(D)** for 6 h and levels of the indicated autophagy markers were analyzed by Western blot (top panels) and relative values were plotted (bottom panels, n = 3). **(F)** Mitochondria are normally degraded in Rnd3 KO cells. Cells were treated with FCCP to induce mitochondria depolarization and/or with hydroxychloroquine (HCQ) to inhibit mitochondrial degradation. Cells were then stained with MTGreen and analyzed by flow cytometry as described in Methods. Mitophagy flux is plotted (n = 3) as the mean fluorescence intensity. UT, untreated. In all experiments ANOVA analysis showed no differences between Rnd3 WT and Rnd3 KO cells.

To get further insight into the function of Rnd3, we used the CRISPR/Cas9 system to generate an NIH 3T3 mouse fibroblasts-derived cell line deficient in the expression of the protein (Figures 2A,B). As we reported previously using other models (Hernández-Sánchez et al., 2015), growth rate of Rnd3 deficient cells (Rnd3 KO) is similar to that of wild-type cells in culture (Figure 2C). We also analyzed autophagy by measuring the autophagic flux in these cells upon serum deprivation (Figure 2D) and looking at different markers by Western blot in extracts from Rnd3 WT and KO cells (Figure 2E). Our results show that Rnd3 KO and control cells have comparable levels of basal autophagy, identified as the degradation of the autophagic protein LC3-II in lysosomes, and similar ability to induce autophagy upon serum removal. Comparison of changes in LC3-II abundance at two different times after inhibition of lysosomal proteolysis also confirmed no differences in autophagosome biogenesis between both cell types. Analysis of the lysosomal degradation of the autophagy adaptor p62 further confirmed that both the basal degradation of p62 and the observed increase in its degradation after serum removal were of comparable magnitude in Rnd3 WT and KO cells (Figure 2E). Moreover, there is no difference in the expression levels of essential macroautophagy effectors such as Atg5, and of the endo-lysosomal marker LAMP2A or the autophagy-related chaperone Hsc70. We also looked at the mitophagic flux to determine whether the presence of irregular mitochondria in Rnd3 deficient mice could be a consequence of problems with the degradation of these organelles. We used the uncoupler FCCP to induce mitochondrial damage and hydroxychloroquine (HCQ) to inhibit lysosomal degradation, to then stain the cells with MTGreen and analyze the mitochondrial mass by flow cytometry. Mitochondria degradation, visualized in these assays as an increase in mitochondrial mass after inhibition of lysosomal proteolysis with hydroxychloroquine, was detected in both Rnd3 WT and Rnd3 KO cells. In fact, mitophagic flux was consistently elevated in Rnd3 KO cells (Figure 2F). All these results indicate that lack of Rnd3 expression does not result in the impairment of autophagy or mitophagy.

**FIGURE 3:**
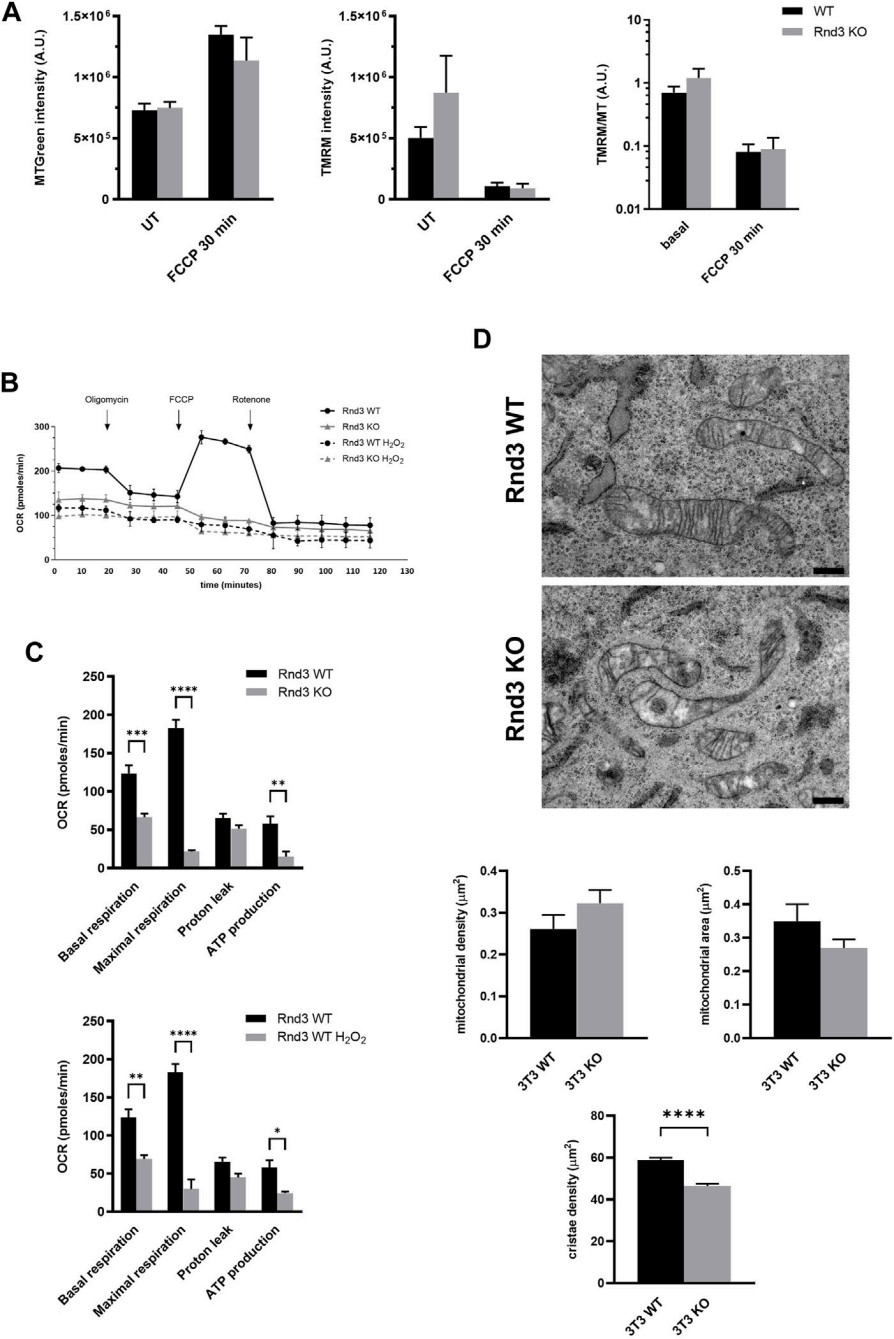
Mitochondrial respiration is impaired in cells lacking Rnd3 expression. **(A)** Mitochondria membrane potential was analyzed by flow cytometry after staining with MTGreen and TMRM, as described in Methods, and the mean values from 3 independent experiments (log scale) are plotted. ANOVA shows no differences between genotypes. **(B)** Seahorse analysis of mitochondrial metabolism reveals an impaired mitochondrial respiration in Rnd3 KO cells. Oxygen consumption rate (OCR) was measured as described in Methods after sequentially adding Oligomycin, FCCP and Rotenone to Rnd3 WT and Rnd3 KO cells and to Rnd3 WT cells treated with 750 μM H_2_O_2_ for 3 h. **(C)** Metabolic analysis of OCR. Data from three independent experiments are plotted. ANOVA shows a significant difference between genotypes (*p* < 0.0001) and no differences between H_2_O_2_ Rnd3 WT cells and untreated Rnd3 KO cells. Bonferroni’s post hoc test shows that Rnd3 KO cells have a reduced OCR in basal respiration (*p* = 0.0002), maximal respiration capacity (*p* < 0.0001) and ATP production (*p* = 0.0030), compared to Rnd3 WT cells. **(D)** Electron microscopy images show irregular mitochondria with a lower number of cristae in 3T3 Rnd3 KO cells (top micrographs). Scale bar: 500 nm. Number of mitochondria was quantified as described in Methods. Mitochondrial density represents the number of mitochondria per cell area. Mitochondrial area was calculated as the average area of mitochondria per cell. Cristae density corresponds to the number of cristae per mitochondrial area. Plots represent the mean and standard error of the mean (SEM) of three independent cultures, 10 cells per culture. Kolmogorov-Smirnov *t* test shows statistical differences in the cristae density (*p* < 0.0001).

We then decided to study the possible role of Rnd3 in the function of mitochondria. We used TMRM to measure the integrity of the mitochondrial inner membrane by flow cytometry, and MTGreen as a reporter for the total mitochondria mass. Cells were treated with FCCP as a positive depolarization control. Lack of Rnd3 expression has no effect on the integrity of the mitochondrial membrane, as there are no differences in membrane potential between Rnd3 WT and KO cells, not even when depolarization is induced with FCCP (Figure 3A). Using a Seahorse analyzer, we have investigated also the mitochondrial oxygen consumption rate (OCR) in Rnd3 WT and KO cells (Figures 3B,C). Our results show that Rnd3 KO cells consume less oxygen (basal respiration) than WT cells. Also, there is a reduction in the OCR associated with ATP synthesis, indicating that these mitochondria are metabolically less active than in WT cells. However, this is not a result of defective membranes, as there is no increase in proton leak. Finally, when Rnd3 KO cells were treated with FCCP, we did not observe the OCR increase characteristic of uncoupled respiration.

We also analyzed these cells by electron microscopy (Figure 3D). Our results show that mitochondria in Rnd3 KO cells are more abundant than in WT cells and that they are smaller, as previously seen in samples from mutant mice (see Figure 1). More importantly, mitochondria in Rnd3 KO cells have a significant decrease in cristae density (Figure 3D, bottom panel). In addition, we obtained embryonic fibroblasts (MEF) from wild type and Rnd3 deficient mice and analyzed them also by electron microscopy, obtaining similar results (Supplemental Figure S2).

All these results suggest that mitochondrial respiration is impaired in the absence of Rnd3 expression, even though the mitochondrial membranes seem to be functional.

It has been shown that oxidative stress can cause an impairment of mitochondrial oxidative phosphorylation (Youle and van Der Bliek, 2012). Treatment of Rnd3 WT cells with H_2_O_2_, led to a pronounced decrease in the OCR associated to ATP synthesis, showing an identical profile to that observed in Rnd3 KO cells even in the absence of H_2_O_2_ (Figures 3B,C). ANOVA analysis shows that there are no differences in OCR between H_2_O_2_ treated Rnd3 WT cells and untreated Rnd3 KO cells, suggesting that the absence of Rnd3 expression could cause an increase in oxidative damage. In fact, H_2_O_2_ treatment causes a decrease in Rnd3 protein levels, which is independent of autophagy (Supplemental Figure S3).

**FIGURE 4:**
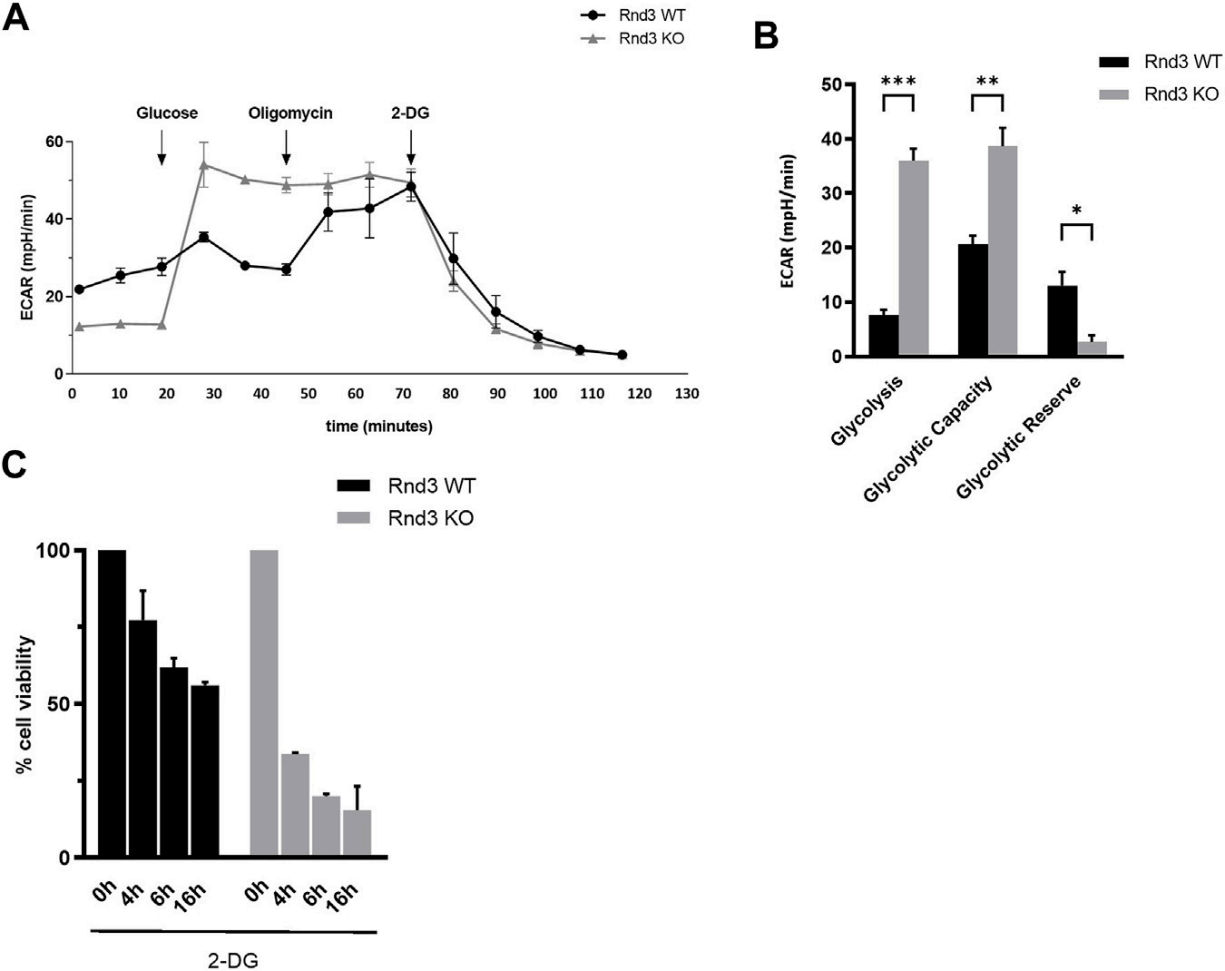
Rnd3 KO cells are strictly glycolysis dependent. **(A)** Extracellular acidification rate (ECAR) was measured in a Seahorse analyzer after sequentially treating Rnd3 WT and Rnd3 KO cells with Glucose, Oligomycin and 2-deoxy-D-glucose (2-DG), as described in Methods. **(B)** ECAR data was used to analyze glycolysis metabolism (n = 3). Glycolysis was calculated as the difference between ECAR following the injection of glucose, and the basal ECAR value. Glycolytic capacity was calculated as the difference between ECAR following the injection of oligomycin, and the basal ECAR value. Glycolytic reserve was calculated as the difference between glycolytic capacity and glycolysis. ANOVA and a Bonferroni’s test show that the difference in behavior of the 2 genotypes is statistically significant (*p* = 0.0005), and that Rnd3 KO cells have increased glycolysis (*p* = 0.0002) and glycolytic capacity (*p* = 0.0030) and decreased glycolytic reserve (*p* = 0.0419). **(C)** Rnd3 WT and Rnd3 KO cell viability was analyzed in the presence of 50 mM 2-DG. ANOVA and a Bonferroni’s post-hoc test show that Rnd3 KO cells are overall more sensitive to glycolysis inhibition than Rnd3 WT (*p* < 0.001) and that there are differences between Rnd3 WT and KO cells at 4, 6 and 16 h (*p* = 0.0035, *p* = 0.0047 and *p* = 0.0048, respectively).

Mitochondrial oxidative phosphorylation is the main ATP synthesis pathway in cells. In its absence, glycolysis becomes the main source of energy. Therefore, we measured the extracellular acidification rate (ECAR), to determine whether glycolysis was more active in Rnd3 KO cells (Figures 4A,B). ECAR increased when glucose was provided to cells, but the difference with the basal ECAR was higher in Rnd3 KO cells than in WT cells. When oligomycin was added and ATP synthase was inhibited, glycolysis was induced in Rnd3 WT cells, as ECAR increased. However, ECAR in Rnd3 KO cells was not affected by oligomycin, confirming that mitochondrial metabolism is impaired in these cells. Final addition of the glycolysis inhibitor 2-deoxy-D-glucose (2-DG) reduced ECAR in Rnd3 WT and KO cells. The analysis of these results shows that Rnd3 KO cells have a higher glycolysis and glycolytic capacity and a lower glycolysis reserve, measured as the difference between glycolysis and glycolytic reserve, than Rnd3 WT cells (Figure 4B).

These results indicate that Rnd3 KO cells are independent of mitochondria metabolism and are using glycolysis as the main source of ATP. To confirm this hypothesis, we measured cell survival in the presence of the glycolysis inhibitor 2-DG (Figure 4C). Rnd3 WT cells showed a moderate decrease in cell viability when glycolysis is inhibited (77%, 61% and 56% at 4, 6 and 16 h respectively). However, Rnd3 KO cells were much more sensitive to glycolysis inhibition, with a dramatic decrease in cell viability (34%, 20% and 16% at 4, 6 and 16 h respectively). These results demonstrate that Rnd3 is relevant for mitochondrial metabolism and that cells lacking Rnd3 expression are strictly dependent on glycolysis to obtain energy.

## Discussion

Rnd3 has been demonstrated to be an essential protein in the CNS development and suggested to be involved in some neurodegenerative diseases (Jie et al., 2015). In this study we reveal an essential role of Rnd3 on mitochondrial function, showing that its absence produces an accumulation of lysosomes, lipid droplets and irregular mitochondria. Furthermore, fibroblasts lacking Rnd3 expression display impaired mitochondrial oxidative metabolism and become strictly dependent on glycolysis. Altogether, these results point out that Rnd3 is crucial for mitochondrial homeostasis, being a key point in neurodegenerative disorders.

We have previously described that mice lacking Rnd3 expression show defects in the development of the CNS (Mocholi et al., 2011; Ballester-Lurbe et al., 2015; Kaur et al., 2020; Madrigal et al., 2021). We now describe some subcellular phenotypes, such as increased content of lipid droplets and accumulation of mitochondria with irregular morphology, that might be compatible with defects in autophagy or mitophagy, as those described in neurodegenerative diseases like Alzheimer’s, Parkinson’s, Huntington’s diseases and amyotrophic lateral sclerosis (Xu et al., 2021). Biogenesis and fusion of membranes are required in autophagy, and regulators of the actin cytoskeleton dynamics have been shown to be involved in this process (Kast and Dominguez, 2017). RhoA acts as a positive regulator of autophagy through its downstream effectors, mainly ROCK-I (Aguilera et al., 2012). However, ROCK-I may act as a positive or negative regulator of autophagy (Gurkar et al., 2013; Mleczak et al., 2013). As Rnd3 is a negative regulator of the RhoA/ROCK-I pathway (Jie et al., 2015), it has been reported that Rnd3 could be modulating the role of ROCK-I on autophagy upon starvation and that it is related to CMA regulated proliferation (Zhou et al., 2016). However, we have not seen any effect of the lack of Rnd3 expression on different autophagy and CMA markers, suggesting that Rnd3 does not play a role in autophagy. Actually, the accumulation of lipid droplets we observe in Rnd3 deficient mouse brains could be a consequence of the severe compromise in mitochondrial oxidation that we describe in Rnd3 KO cells.

We also found that mitochondria turnover is not impaired in cells lacking Rnd3 expression. In fact, we detected a consistent elevated mitophagy flux in Rnd3 KO cells, supporting that the observed mitochondrial alterations are not secondary to their impaired turnover, but rather a primary defect, and that the observed enhanced mitochondrial degradation in Rnd3 KO cells could be a defense mechanism activated to reduce aberrant mitochondria accumulation.

Although new evidences are emerging about the regulatory function of mitochondria and cell metabolism during neurogenesis (Bueler, 2021), the involvement of Rho proteins in mitochondria homeostasis is still unclear: ROCK-I downregulates Parkin mediated mitophagy in PD (Moskal et al., 2020); on the other hand, activation of the RhoA/ROCK-I pathway results in the phosphorylation of Dynamin-related protein 1 (Drp1) which leads to mitochondria fission in cardiomyocytes under stress (Brand et al., 2018). ROCK-I activity also mediates high glucose stress induced mitochondrial fission and dysfunction, leading to an increase of mitochondrial fragmentation, ROS generation and a decrease of ATP production (Chen et al., 2019). Rac1 is also localized in mitochondria and its inhibition has a neuroprotective role through mitochondrial regulation, reducing the oxidative stress (Osborn-Heaford et al., 2012; Natsvlishvili et al., 2015; Pan et al., 2018). Although Rac and Rnd proteins share homology, it has been demonstrated that they play different roles during neural development control (Negishi and Katoh, 2002; Ishikawa et al., 2003). Therefore, the role of Rho proteins, and particularly Rnd3, in mitochondria homeostasis needs to be further clarified.

Different stressors, including high glucose and ROS generation, induce mitochondria fission and inhibit oxidative phosphorylation, which contributes to several neurodegenerative disorders (Rossmann et al., 2021). As Rnd3 is an inhibitor of the RhoA/ROCK-I/MLC pathway, Rnd3 deficient mice show an increased activity of this pathway (Peris et al., 2012). As such, Rnd3 could play a role in mitochondria fission under stress conditions. In fact, Rnd3 expression is reduced in trophoblastic cells under hypoxia, which induces an increase in ROS and a decrease of PPAR-γ and UCP2 levels, resulting in cell apoptosis and mitochondrial injury (Huang et al., 2021). Moreover, a recent study shows that Rnd3 overexpression attenuates oxidative stress in spontaneously hypertensive rats through ROCK1 inhibition (Wu et al., 2021). Although we did not analyze superoxide levels, we also see a decrease in Rnd3 expression in cells treated with H_2_O_2_ and the accumulation of mitochondria with a morphology compatible with fission in Rnd3 deficient mice. In fact, OCR is not affected by oligomycin nor by FCCP in the absence of Rnd3 expression or when cells are treated with H_2_O_2_. This is not a result of an increased proton leak, as it is not different from wild type cells, but may be due to a lack of electrons in the respiratory chain: mitochondrial ATP synthesis seems to be inhibited and OCR does not increase when mitochondria are uncoupled with FCCP. Although the reduced number of mitochondria cristae in Rnd3 KO cells could be causal to the impaired oxidative metabolism (Cogliati et al., 2016), further studies are required to determine whether the respiratory chain complexes or the Citric Acid Cycle components may also be affected by the lack of Rnd3 expression. In fact, as Rnd3 is not found in mitochondria enriched subcellular fraction, the effects we see might be indirect. We propose that Rnd3 acts as a regulator of the RhoA/ROCK-I pathway in such a way that, when cells are under stress, downregulation of Rnd3 expression allows the induction of mitochondria fission by the RhoA/ROCK-I pathway which, in turn, inhibits oxidative phosphorylation and activates glycolysis. Also, the severe compromise in mitochondria β-oxidation in absence of Rnd3 could be responsible for the accumulation of lipid droplets in the Rnd3 defective brains.

Altogether, our results suggest that Rnd3 is relevant to maintain mitochondria homeostasis and ATP synthesis through oxidative phosphorylation. Lack of ATP synthesis could explain the phenotypes we observed in mice lacking Rnd3 expression including smaller size, early death and defects in CNS development (Mocholi et al., 2011). To notice, it has been reported that differentiation of stem cells into mature cells requires a switch from glycolysis to increased mitochondrial respiration (Chen et al., 2008; Maryanovich et al., 2015) and having in mind the role of defective mitochondria homeostasis in neurodegenerative diseases, our findings point at Rnd3 as a relevant player in maintaining CNS functions. It is therefore tempting to suggest that Rnd3 activity could be important in preventing the onset or delay progression of these diseases. Further studies will be needed to clarify the actual role of Rnd3 in mitochondria metabolism and its relationship with neurodegenerative diseases.

## Data Availability

The original contributions presented in the study are included in the article/Supplementary Material, further inquiries can be directed to the corresponding authors.
