# Supplementary figures and images for: Rnd3 Expression is Necessary to Maintain Mitochondrial Homeostasis but Dispensable for Autophagy
Source: Front Cell Dev Biol. 2022 Jun 27;10:834561. doi: 10.3389/fcell.2022.834561 (PMC9271580; doi:10.3389/fcell.2022.834561)

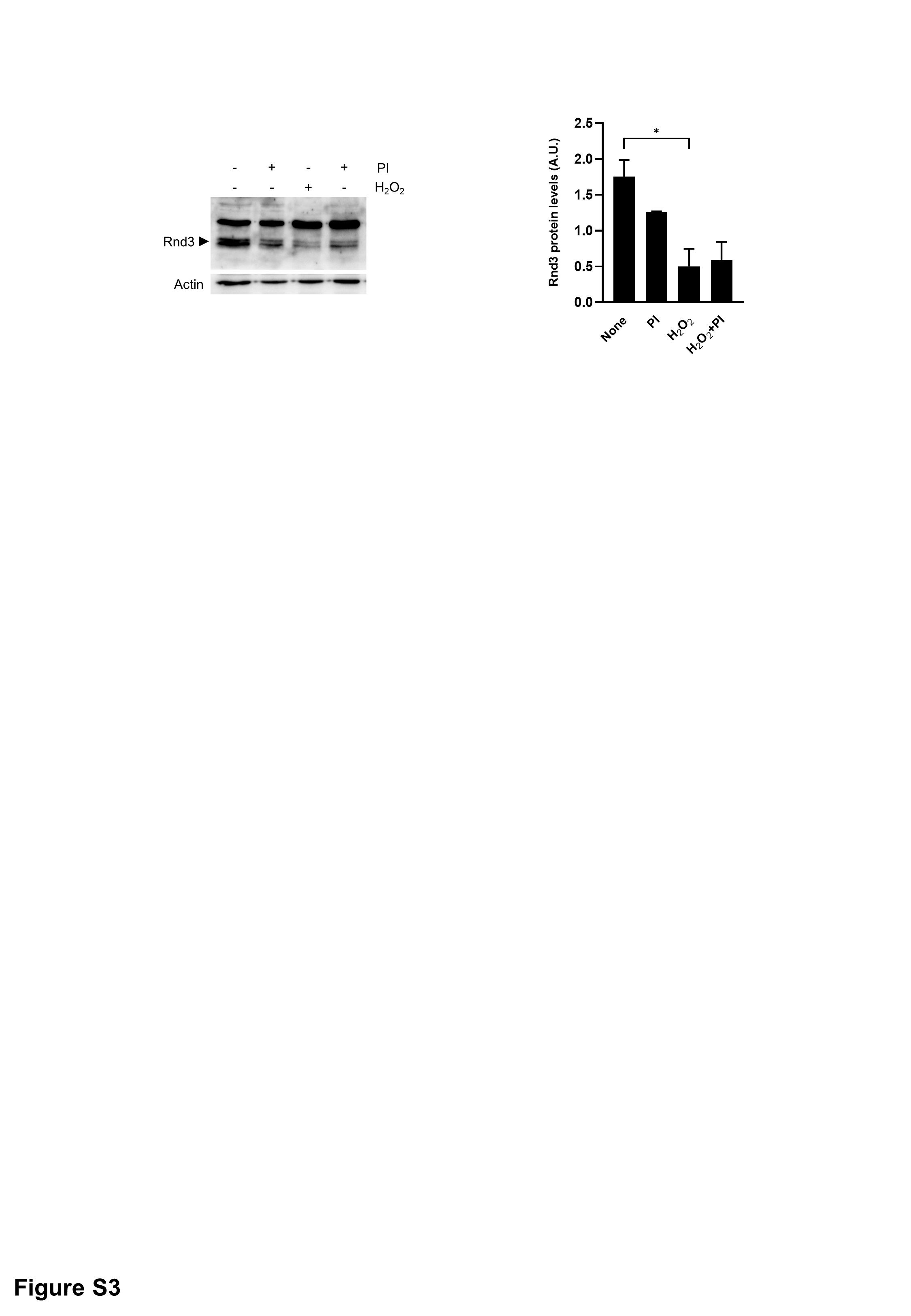

Supplement: Supplementary file 1 [file Image3.jpeg]

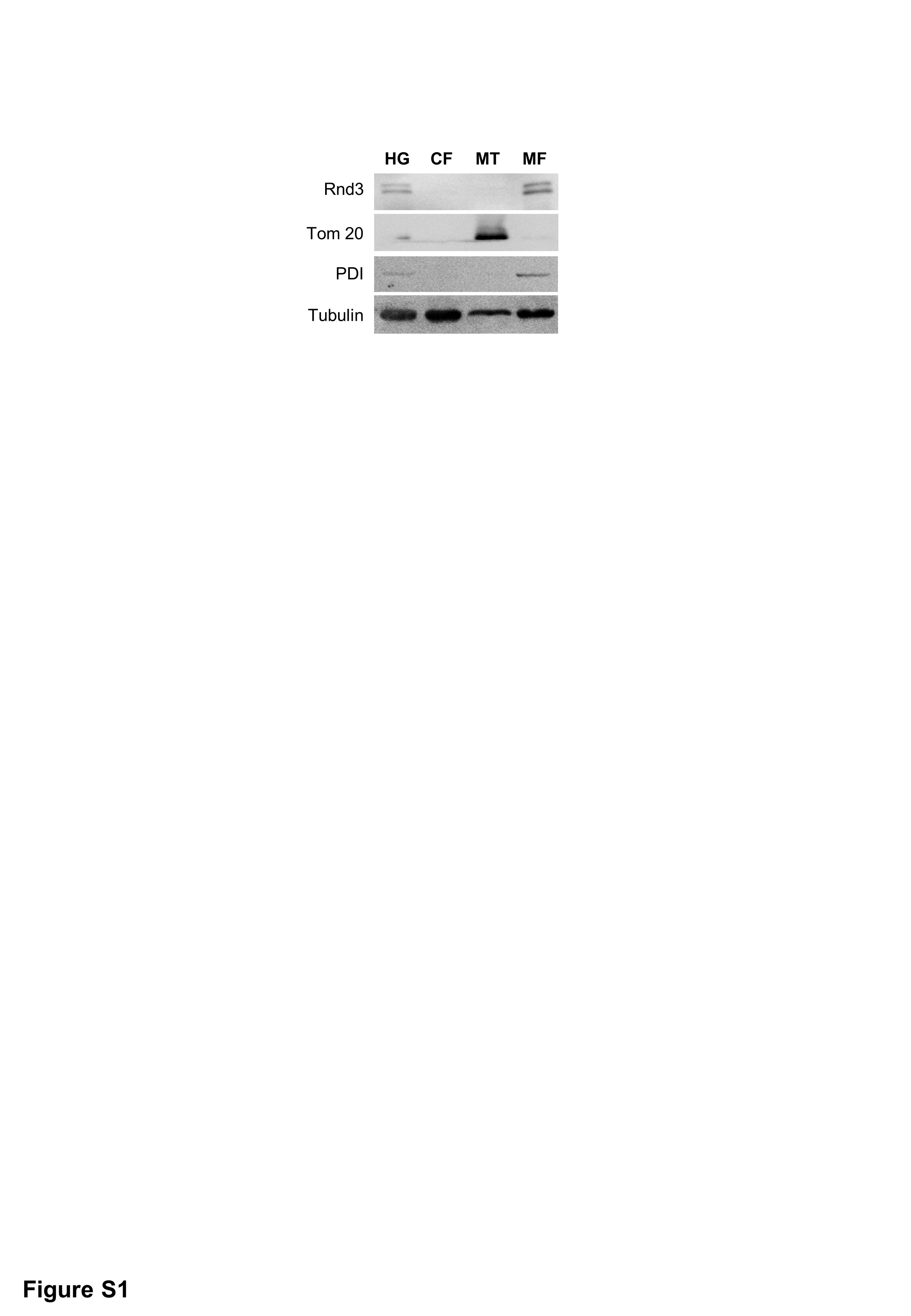

Supplement: Supplementary file 2 [file Image1.jpeg]

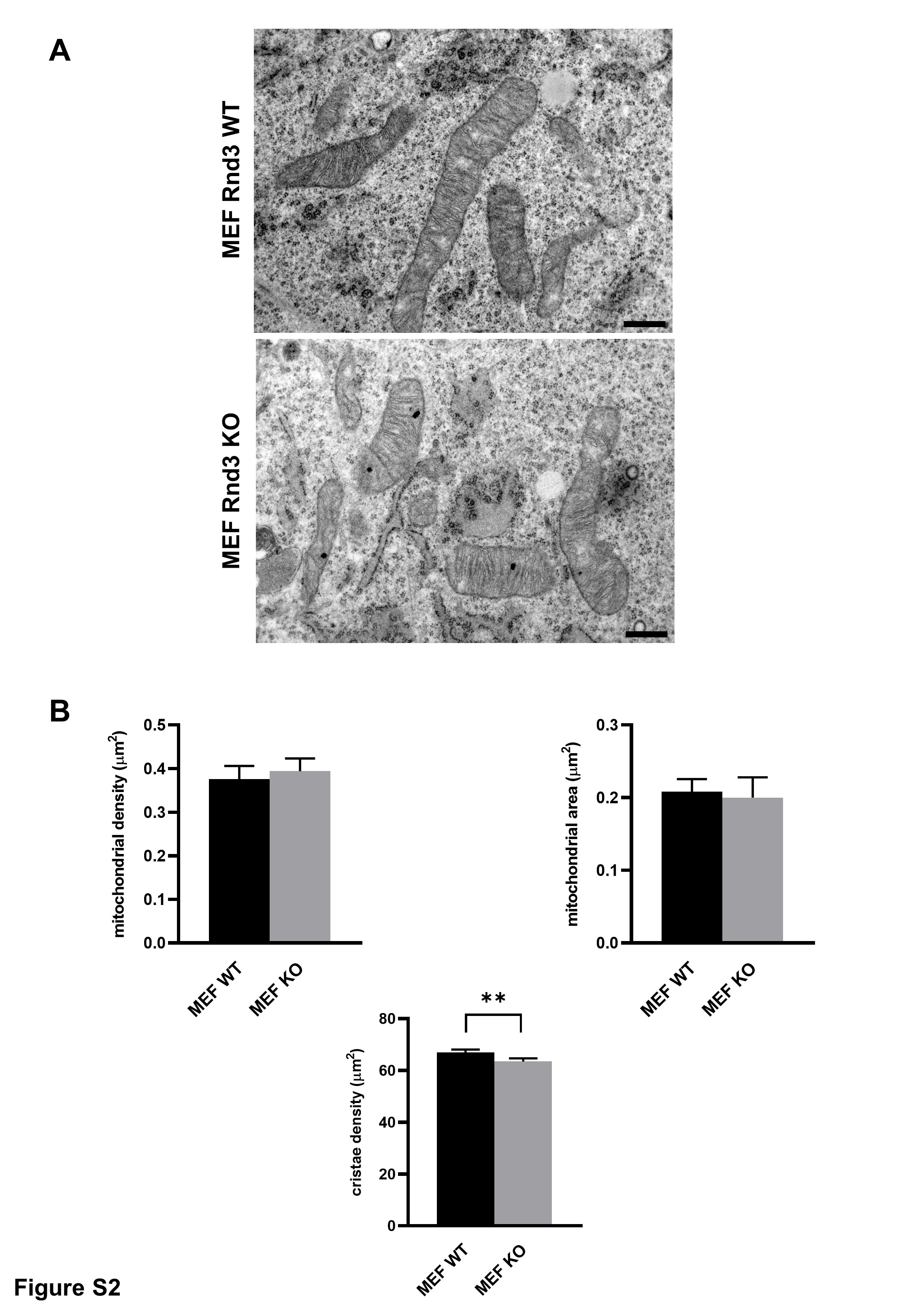

Supplement: Supplementary file 3 [file Image2.jpeg]
